# TF4SM: A Framework for Developing Traceability Solutions in Small Manufacturing Companies

**DOI:** 10.3390/s151129478

**Published:** 2015-11-20

**Authors:** Borja Bordel Sánchez, Ramón Alcarria, Diego Martín, Tomás Robles

**Affiliations:** 1 Departamento de Ingeniería de Sistemas Telemáticos, Universidad Politécnica de Madrid, Avenida Complutense n°30, Madrid 28040, Spain; E-Mails: diego.martin.de.andres@upm.es (D.M.); tomas.robles@upm.es (T.R.); 2 Departamento de Ingeniería Topográfica y Cartografía, Universidad Politécnica de Madrid, UPM Campus Sur, Km 7.5 de la Autovía de Valencia, Madrid 28031, Spain; E-Mail: ramon.alcarria@upm.es

**Keywords:** traceability, real-time manufacturing, manufacturing execution system, cybernetic devices, cybernetic glove, cybernetic table, prosumer, RFID, Bluetooth

## Abstract

Nowadays, manufacturing processes have become highly complex. Besides, more and more, governmental institutions require companies to implement systems to trace a product’s life (especially for foods, clinical materials or similar items). In this paper, we propose a new framework, based on cyber-physical systems, for developing traceability systems in small manufacturing companies (which because of their size cannot implement other commercial products). We propose a general theoretical framework, study the requirements of these companies in relation to traceability systems, propose a reference architecture based on both previous elements and build the first minimum functional prototype, to compare our solution to a traditional tag-based traceability system. Results show that our system reduces the number of inefficiencies and reaction time.

## 1. Introduction

In recent years, companies, government institutions, researchers and traders, among others, have stressed the importance of manufacturing process monitoring systems as a primary source of information. Projects such as [1] or works such as [2,3] demonstrate the importance of the ability of systems to extract valuable data from these processes. However, as the complexity of manufacturing processes increases, monitoring (especially if involving human operators) becomes very costly and inefficient [4]. From the beginning, solutions based on information technologies were adopted to reduce the complexity associated with traceability by operators. Relevant works, such as [5,6,7], have developed successfully traceability solutions categorized as “Smart Environments” or “Wireless Sensor Networks”. Moreover, approximately between 2000 and 2010, a number of important manufacturing companies (specially leading vehicle manufacturers) started to employ radio frequency identification (RFID) to facilitate traceability in manufacturing processes. First, Volvo Trucks established a RFID system to achieve continuous production [8,9]. Later, Toyota implemented information systems based on RFID to track auto parts production [10,11]. Most recently, in 2012, the Chinese manufacturer Guangdong Greatoo Molds Inc. deployed a RFID-enabled manufacturing execution system as a case study [12].

However, the previous examples relate to large-sized companies, while most manufacturers are actually small and medium-sized companies, which must face financial and technological challenges when they attempt to implement RFID-based traceability systems. Currently, most of the RFID systems are provided by important information technology companies, such as IBM or Ubisense. The kind of solutions developed by these Information Technologies (IT) providers makes the cost and technical requirements of adopting their RFID solutions too high for small and medium-sized manufacturing companies [13,14]. When a small manufacturing company considers applying an automated traceability system (particularly a RFID-based system) three basic questions must be confronted [12]. The first is how to deploy RFID devices to collect real-time data in a cost-effective way. The second is how to translate the real-time data into meaningful information to enable the field operator’s convenient operations (e.g., inefficiency detection and correction). The third is how to integrate the RFID system into the company’s current production processes.

We argue herein that a framework strongly based on Cyber-Physical Systems (CPS) can successfully address all the previous questions. As described in the relevant NSF reports [15] CPS are integrations of computation and physical processes. Embedded computers and networks monitor and control the physical processes, usually with feedback loops where physical processes affect computations and *vice versa*. However, this initial definition has been modified and expanded by many authors. If in the first instance CPS referred to a kind of networked embedded systems running real-time applications, nowadays CPS (as described by the National Institute of Standards and Technology [16]) have a more vertical approach, including from the user goals to the underlying physical system. In addition, in this second focus, issues related to traceability systems, such as the Human-System Interaction, are a main part of the architecture [17].

Notwithstanding this great interest, the heterogeneity in CPS has revealed several problems in industrial applications that cannot be easily solved through current control, communications, and software theory [18]. Consequently, some industrial researchers have recently returned to the simplicity and uniformity of Wireless Sensor Networks (WSNs) [19] to integrate them with traditional intelligent industrial systems. Thus, new definitions, such as Industrial Wireless Sensor Networks [20] have appeared. Nonetheless, the knowledge created around Cyber-Physical Systems could provide many advantages over existing industrial applications [18].

In this paper we propose a framework based on CPS to reduce the complexity associated with its application in manufacturing scenarios, including other aspects originally derived from traditional intelligent industrial solutions. The objective of this paper is to describe this framework, and to validate its usability as a traceability solution for reducing the number of inefficiencies and to improve their time response in companies. Thus, the contribution of this paper is the proposal of a CPS-based traceability framework, merging both CPS technologies and traceability capabilities. Our work also includes a validation of how a proof of concept of this framework can reduce inefficiencies and improve time response for industrial processes in companies.

In order to validate the Traceability Framework for Small Manufacturers (TF4SM) as a useful framework to develop traceability systems and applications, the authors conducted an experimental validation to address the following two research questions:

1. Would the time response to inefficiencies improve by deploying a system based on our TF4SM in companies?
2. Is it possible to reduce the number of inefficiencies in productive processes using a TF4SM-based system?

The experimental validation consisted in two typical kinds of manufacturing processes: logistical shortages detection process in warehouses and productive activities that lead to a quality system checkpoint. A statistical analysis of the results showed that a system based on our TF4SM provides a significant improvement in time response to inefficiencies in companies and a remarkable reduction in the number of inefficiencies due to procedural errors.

The rest of the paper is organized as follows: Section 2 introduces the state of the art in traceability systems and automated industrial processes, as well as different theories about networked embedded systems and related commercial products. Section 3 analyzes the requirements of small manufacturers, presents the reference architecture of our new framework and the design of the first minimum functional prototype based on it. Section 4 describes our proposal hardware implementation and our proposed process model. Section 5 provides an experimental validation of this approach. Finally, Section 6 and Section 7 explain some results of this experimental validation and the conclusions of our work.

## 2. State of the Art in Traceability Systems, Different Theories of Networked Embedded Systems, and RFID-Based Products

During the last fifteen years, many concepts referring to networked embedded systems have appeared. As mentioned in the Introduction, some of them have already been used as a framework for building traceability solutions. However, we argue that the CPS paradigm fits better with traceability solutions than any other previous definition.

In this Section we review the state of the art in traceability solutions and automated industrial processes. Later, we analyze different definitions of networked embedded systems to find the one which best fits with traceability systems. Finally, a review of RFID integration in daily living objects is provided.

### 2.1. Automated Industrial Processes and Traceability Systems: State of the Art

The application of software engineering to industrial automation spans from manufacturing automation to process control systems and energy automation systems. There is ample evidence of the extensive use of automated systems in industrial environments. For example, the German Engineering Federation has calculated [21] the ratio of control software to the cost of machinery where it is used has doubled in value from 20% to 40% in one decade.

The Automation Research Corporation (ARC) advisory group [22] and the International Electrotechnical Commission (IEC) [23] distinguish various kinds of automation products for industrial processes, among which we find the high and low power AC drivers, human machine interfaces (HMIs) and programmable logic controllers (PLC). In most solutions, several of these products must be integrated creating libraries for the interconnection which, in most cases, are proprietary technologies [24]. Thus, typically, there is not a homogeneous solution that could be standardized (except for certain cases, such as Object Linking and Embedding—OLE—for Process Control, usually called OPC [25]). Therefore, our approach should be a complete integrated solution, which would include all the necessary functions for automated industrial processes. This approach would allow a reduction in the system’s complexity, control the investment needed, remove technical positions and, ultimately, reduce the cost of deploying and operating systems [26]. In that way, we could address the first basic question presented in the Introduction (“how to deploy RFID devices to collect real-time data in a cost-effective way?”).

Later, nowadays, most components automated industrial process solutions virtualized, however, the structure is similar to that of the traditional hardware architecture [27]. In these systems, there are four types of devices communicating via networks: an engineering and simulation station (including a database and a programming software), a human-machine interface (HMI), at least one programmable logic controller (PLC) with control software and, at least, one driver controlling machines. It would be a good practice to adapt the TF4SM-based system to this generic structure (already tested in industry).

On the other hand, traditional automated industrial systems require workers to train in both software engineering and industrial processes (as PLC must be programmed in proprietary low-level languages [28,29]). In order to improve the cost efficiency (so important in small companies) a prosumer-oriented [30] framework would be desirable. Thus, only industrial domain experts would be necessary.

Furthermore, the embedded devices usually considered in automated industrial processes (including traceability solutions), implement a fixed network architecture model, making it impossible to monitor workers, replace the devices easily or rearrange the machine distribution [31,32]. This increases the cost of system updates and tracking of manual processes. Then, adaptable policies should be included in traceability systems for small companies, so workers can be monitored without affecting their movement and machines could be replaced, rearranged or removed without modifying the system configuration.

Specifying now the debate on existing traceability systems for small-sized companies, five different technological states coexist in small companies nowadays, namely:

1. There are some manufacturers that do not apply any traceability system, such as small handicraft companies where production processes do not generate intermediate products [33].
2. Some companies implement systems based on invoices and control record sheets (commonly known as “system based on pen and paper” [34]). In these systems, each product carries a label with a serial number and a description. An operator is responsible for, at each stage of the production process, writing both data items in the appropriate form. Although these systems work fine, the use of IT enables a larger amount of data to be handled, and thereby it becomes realistic to develop traceability systems with very detailed information about both the product and its processing history [35,36].
3. We find companies which implement systems based on Personal Digital Assistants (PDAs) [37]. In these systems, each product still carries a label with a serial number and a description; however, in this case the responsible operator, instead of using control record sheets, uses a PDA to transmit data in real-time. Although these systems reduce the time requirements related to traceability processes, they still are extremely sensitive to human errors.
4. Around ten years ago, in order to reduce human errors in traceability systems, labels with the serial number and the description of each product were replaced by codes (e.g., QR or barcodes) storing both data [38]. Then, equipping PDAs with the appropriate reader is enough to avoid operators having to manually input any data.
5. More recently, the old stickers with printed codes have been replaced by RFID labels [39]. This is basically due to two factors [40]: RFID tags can store kilobytes of information, and they also can include security and privacy policies (what it is especially important in confidential products). These systems have been widely applied in the manufacturing sector.

Our framework intends to improve current traceability systems in such a way that new systems would not require performing traceability-specific tasks; instead, traceability information can be deduced from the usual activities of operators.

### 2.2. Networked Embedded Systems and Traceability Systems

As stated in the previous section, we need a complete integrated solution to collect data in real-time in the most cost-effective way. Moreover, the framework must include adaptation capabilities and allow the development of system architectures similar to the generic structure of automated industrial processes. Finally, if product life cycle has to be deduced from the usual activities of operators, RFID readers (we selected RFID as the base technology for its successful results in previous deployments) must be seamlessly integrated into daily objects.

Given these premises, it is necessary to first select a basic theory that will help us develop our framework. It is clear that this basis must be related to networked embedded systems. The first efforts for integrating information technologies in the physical world and supporting different applications (including traceability systems), occurred in the electronics world. Since 2000, several terms have appeared in the literature and have been proposed in conferences to describe smart distributed electronic systems (more or less embedded in the physical world): Smart Home [41,42], Smart Office [43], Intelligent Home [44], and Smart Environments [5,45,46], among others.

In parallel, some of the existing definitions delimited what kind of infrastructure could be considered one of these new systems. In 2000, [7] stated that: “a smart space is a region of the real world that is extensively equipped with sensors, actuators and computing components”. In 2002, some authors decided to propose their own definitions. [47] defines a Smart Environment as: “a system that is able to autonomously acquire and apply knowledge about the environment and adapt to its inhabitants’ preferences and requirements in order to improve their experience”. In 2005, the same authors proposed in [5] a new definition: “a smart environment is a physical world that is interconnected through a continuous network abundantly and invisibly with sensors, actuators and computational units, embedded seamlessly in the everyday objects of our lives”.

Around 2010 interest in these systems waned, and researchers abandoned attempts to formalize the theoretical framework. At that time, a new concept had captured everyone’s attention: cyber-physical systems (CPS) [48]. The term “cyber-physical systems” emerged around 2006, when it was coined by Gill at the National Science Foundation in the United States. Later, Lee developed the term in a relevant NSF report [15]. However, in this work CPS are described as networked entities for real-time processing. Thus, research challenges are related to computational problems such as the creation of real-time applications or operating systems [49]. Meanwhile the interest in CPS has changed to more vertical issues such as networking, communications, data services, decision making and pattern recognition.

**Figure 1:**
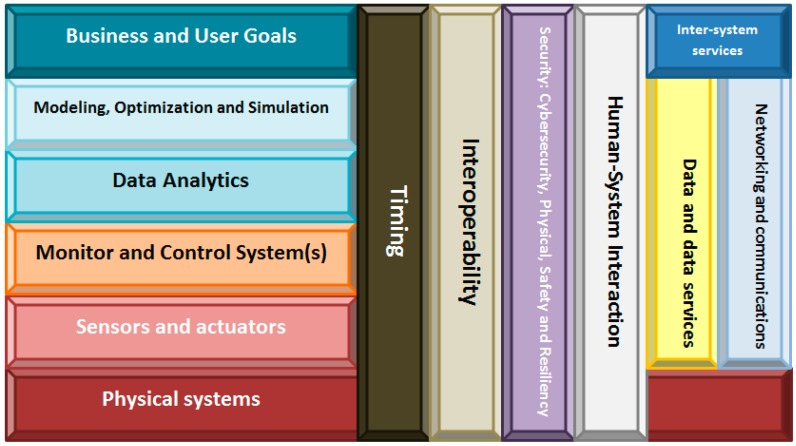
Reference CPS architecture (NIST).

Extremely important is the reference architecture (see Figure 1) proposed in June 2015 by the National Institute of Standards and Technology (NIST). The CPS public working group proposes that: “cyber-physical Systems or “smart” systems are co-engineered interacting networks of physical and computational components”. Besides being more general than the original, this new definition and reference architecture present the advantage of being the standard on which commercial products of CPS will be based.

Table 1 compares the most important theories about networked embedded devices in relation to the classical features of RFID-based traceability systems (as described in [49]). Besides, the requirements deducted in the previous sections related to the three basic questions about traceability systems in small manufacturers (see the Introduction) are also included. As can be seen, CPS (as defined by the NIST) are aligned to the requirements of traceability systems, so we selected its reference architecture as the basis for our framework.

**Table 1:** Comparison among different theories about networked embedded systems in relation to traceability systems.

Feature and Requirements	Smart Space Along [7]	Smart Environments Along [1]	Smart Environments Along [5]	CPS From [15]	CPS From NIST
Object identification capability	✓/✗	✗	✗	✓	✓/✗
Real-time data capture	✓	✓	✓	✓	✓
Execution engine	✗	✗	✗	✗	✓/✗
Decision capability	✗	✗	✗	✓/✗	✓
Learning capability	✗	✓	✗	✓/✗	✓
Prosumer focused	✗	✗	✗	✗	✓/✗
Dynamical adaptation	✓/✗	✓/✗	✓/✗	✓/✗	✓
Vertical approach	✗	✓	✗	✗	✓
Can offer meaningful human-understandable information	✗	✓	✓/✗	✓/✗	✓

✓: Included in the definition; ✗: Not considered in the definition; ✓/✗: Available in some works.

In the last three years, various works relating to CPS and process monitoring and traceability systems have appeared. In [50], the future demands on automation systems (based on CPS) are shown, and new strategies for software deployment of automation applications and communication systems are discussed; more specifically, the system presented in [51] describes an industrial cloud-based CPS.

Some works, such as [52], focused on designing communication models for monitoring systems and processes using CPS while others provide simulation environments for CPS-based industrial systems ([53] describes a CPS research project to support virtual design and verification of industrial processes).

However, as we mentioned in the Introduction and can be seen in [18], the actual deployment of CPS in manufacturing environments has several drawbacks that are not easily resolved. We argue the new reference architecture proposed by the NIST will allow us to develop a framework being able to address these issues.

### 2.3. RFID-Based Products

Compared to other research on automated industrial processes and traceability systems (usually focused on simulation environments [54], software compatibility [55,56], wireless sensor networks [20], or safety in automation systems [57]), our framework will not only consider high-capacity generic computing platforms, but also both software and hardware components. Thus, all knowledge of low-energy computing platforms, seamless integration of electronic systems and real-time communications (originating from the world of CPS) can be applied to the manufacturing environment.

In this section, we focus on hardware components and review the state of the art in relation to the integration of RFID tags and readers in different everyday objects. In that way the third basic question about traceability systems in small companies (how to integrate RFID systems into the company’s current production processes?) is answered: by means of embedded tags and readers in daily objects.

Due to the business interest in control and traceability systems, over the years, some products, articles, conferences and so on have been about issues related to our aim (directly or indirectly).

On the one hand, as we have mentioned, since 2000, various research proposals for integrating electronic systems at home or in the workplace emerged: [41] present the concept of “Smart Home”, in [43] a “Smart Office” is designed and an “Intelligent Home” is planned in [44].

Later, between 2003 and 2004, the concept of “smart furniture” became very popular in research. Various research papers on this subject, such as the general framework presented in [58,59], appeared. Furthermore, some patents, such as [60], in which a RFID smart chair is described, were also given.

Finally, related to wearable technologies, several papers describe their use in traceability systems, especially cybernetic gloves. Thus, in 2005 a first prototype of a cybernetic glove appeared [61], and recently, in February 2014, Fujitsu presented its own design for industrials environments [62]. Independently, textile industry advances in the seamless integration between electronics and textiles, will enable a qualitative leap in wearable products [63,64].

On the other hand, although they are not considered integrated systems, several commercial “smart products” for stock control, environment adaptation to user and traceability have been developed recently.

In 2011, a smart poker table was presented in Italy by GTI Gaming [65]. Based on RFID technology, this table implements a real-time technology capable of calculating the amount of pot, and rake, reporting defects, identifying the dealer, establishing a network of tables and so on.

Later, between December 2013 and February 2015, up to three smart clinical medicine dispensers were presented in Europe. The first, in Portugal [66], was developed with Fujitsu’s collaboration. The others [67,68], developed in Spain in June 2015, are still undergoing tests.

Nevertheless, the most successful product in the market is SATO’s VINICITY technology [69]. This technology is able to read various RFID tags at once, so it has been employed in several “smart products” such as trays, tables and medicine dispensers.

## 3. Analysis of Manufacturing Scenarios. TF4SM: First Prototype Design

Manufacturing processes require precise control over each one of the phases comprising a process. Therefore, traceability becomes an increasingly important functionality to be implemented in process control systems. This section analyzes manufacturing scenarios in small companies (the motivation scenario of our work), and the requirements to be met to enable precise monitoring and traceability information acquisition. After describing the requirements of small manufacturing companies, we present the new traceability framework and the design of a first minimum functional prototype is proposed.

### 3.1. Analysis of Manufacturing Scenarios: Motivation Scenario

Manufacturing companies, in their day to day operation, perform various kinds of processes, such as logistic processes (including the receiving process and the inventory process) and production processes. The *receiving process* models the arrival of some units that must be tagged with RFID and reported in the corresponding receipt notice (which it was traditionally printed and, nowadays, it is digitalized). The received units may optionally aggregate into larger logistics units. If some units are not included in a purchase order, typically they must be discarded. After that, the received logistics units are moved to a warehouse. The execution results are reported in the corresponding transfer notice (which, one more time, was traditionally printed and, nowadays, it is digitalized). An *inventory process* is executed in parallel, and applies to all stored items that are cyclically counted. It would be a desirable feature that the system will issue alerts in cases of shortage or inconsistency of inventoried goods.

During the *production process*, warehouse operators select and supply the required quantities of raw materials that are specified in the production task instructions and attach the RFID labels of their containers onto the electronic reader. The production operator attaches his personal identification card to start a *production task*. Once the production process is finished the operator prints new RFID and/or barcode labels (depending on the system implemented) for the remaining materials that are scheduled for return to the warehouse and the products (ready or semi-finished) that are processed during each production task. Traceability information is used to execute consistency checks to determine quantities of raw materials consumed, the spoilage, the utilization of tools and labor, as well as the machine operation and stop times. It would be a desirable feature that the system will issue alarms in case of discrepancy (*i.e.*, illegal operator or equipment), excessive consumption, and/or low production rate.

Taking into account the previous description, some additional requirements (introduced by the specific needs of small-sized manufacturing companies) have been considered in the framework development (apart from the ones presented in the previous sections):

- REQ#1: the event monitoring function and production task traceability must be independent of the location of the information source and the hardware used. Traditional control systems were limited to the capability of the controller residing inside.
- REQ#2: it should define a flexible and extensible architecture for the integration of various systems, wireless sensor networks (WSN), actuators, execution engines, among other things.
- REQ#3: it should be capable of suspending current operations when the data obtained is corrupt, the task performed does not correspond to the expected one and/or some defined rules for the tasks are not satisfied.
- REQ#4: the system must be easily configurable to allow communication with new elements and seamless interchange of new events. Traditional tag-based systems required early binding, that is, a configuration stage to link data sources and consumers of data.
- REQ#5: the infrastructure must be capable of identifying each user or user role to determine the task to be performed. User roles are also considered for the cases when regulation does not allow identification of employees.
- REQ#6: the system must assign tasks to different users who collaborate to complete the process.
- REQ#7: ability to reconstruct every production step. This is required both for regulatory compliance and as an important basis for production environments.

### 3.2. TF4SM: A New Traceability Framework for Small Manufacturing Companies

**Figure 2:**
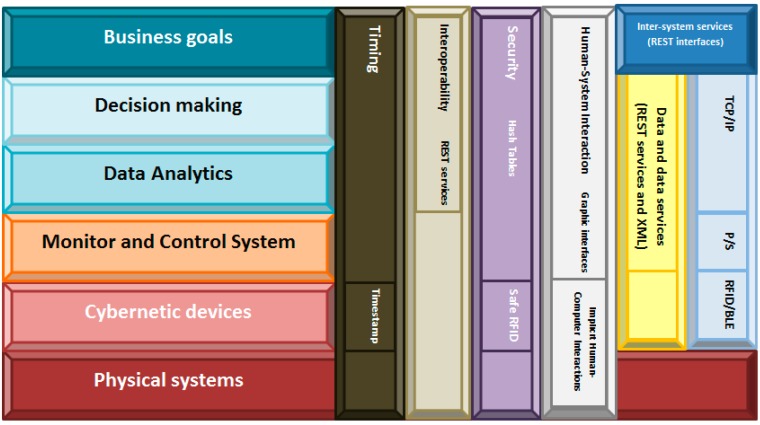
TF4SM architecture.

As we have seen, improving traceability systems in small-sized manufacturing companies inevitably requires using information technologies. Among all the available technologies, we selected cyber-physical systems (and specifically, CPS according to the NIST definition) as the most appropriate. Therefore, we took the reference architecture showed in Figure 1 as our basis. Over it, we added or changed the elements necessary to fully check the requirements described in the previous sections. Our proposed Traceability Framework for Small Manufacturers (TF4SM) is the result of this process (Figure 2).

Next, we are briefly describing each part of the framework, and how they cover the previously presented requirements. The TF4SM framework consists of six levels of abstraction:

- The physical systems include all instruments, industrial systems and manufacturing elements and products present in the company’s facilities.
- Cybernetic devices layer refers to the embedded devices which contains RFID readers and/or RFID tags, as well as to all elements which make up the subjacent pervasive computing system [70]. The inclusion of mobile computing in the framework (we describe the communications plane later) partially covers REQ#1 (the system must show correct performance, independently of the information source’s location). In addition, working with embedded devices allows us to answer the third basic question about traceability systems: how to integrate RFID system into the company’s production processes. Although this paper refers to cybernetic devices, this kind of technologies has been commonly studied as “smart objects”. For example, in [71] the relevance of smart objects is highlighted in several application scenarios.
- The Monitor and Control System refer to the interlayer which adapts cybernetic devices to high level computing schemes. It contains, mainly, data acquisition from RFID readers and data pre-processing. The inclusion of this layer allows creating hardware independent traceability systems. Embedded devices are placed below this layer and knowledge feedback loops are defined on top. This breakdown between knowledge management processes and the hardware which supports the system agree fully with the second part of REQ#1.
- The Data Analytics layer, as in NIST reference architecture, processes data from different devices for pattern recognition and extracts data using machine learning and data mining. It also includes visual analytics oriented to the operator. Moreover, pattern recognition capability fulfills REQ#7 (which describes the ability to reconstruct every production step), as it allows inferring workers’ activities from data collected by RFID readers. The algorithms included in this layer also answer the last basic question which remains unresolved (how to translate the real-time data into meaningful information for the field operator?). Decision making uses results from Data Analytics as input for determining the next action which must be executed (issue an alert, suspend an operation, *etc.*). Thanks to the support of Data Analytics layer corrupt data can be detected, and this layer can order an operation to be suspended. In that way, REQ#3 is covered, and some desirable features are included.
- Finally, in the Business Goals layer, rules about alerts, user’s roles, minimum stock, automatic orders or emergency situations can be defined. The interface offered by the system at this level will be specific of the application domain (depend on the product manufactured in the company), so any technological expert must control the traceability system (what was desirable). As this layer is placed at human abstraction level, identities, workflows, tasks or collaborative work can be defined. Thus, REQ#5 and REQ#6 get fulfilled.
- Apart the previously described layers, the framework architecture includes six planes which cross vertically all the layers:
  - Timing: In traceability systems, time issues are not critical. Therefore, in our framework timing has been resolved including a timestamp in each data produced by the cybernetic devices. Any other solution is considered as this datum is enough for tracing the products’ life;
  - Interoperability: Traceability systems are conceived as corporative infrastructure, so typically any external access will be admitted. In the case of joining various systems, communication will be performed at high level through Representational State Transfer (REST) interfaces, much more flexible than other technologies;
  - Security: Security systems native from RFID-based traceability systems (based on safe RFID tags [72] and hash tables) have been widely proved as enough safe, so we maintain the same technologies in our framework;
  - Human-System interaction: Two different levels are distinguished. At cybernetic devices layer, any direct interface toward the physical world is described, so the communication will be based on Implicit Human-Computer Interaction (IHCI) [73]. In higher layers, specific graphical interfaces will be defined to communicate operators with the system;
  - Data and data services: The same philosophy applied in the case of “Interoperability” is valid here;
  - Networking and communications: Communications must support a flexible architecture [74,75]. In order to achieve this objective four technologies coexist in our proposed framework. First, cybernetic devices must implement RFID technology (to trace products’ life) and Bluetooth Low-Energy (BLE) standard (to transmit the readings toward the monitor and control system). The use of wireless technologies, in addition, supports the creation of a mobile computing scheme. Second, over BLE, a Publish/Subscribe protocol will be deployed. Actually, the use of the Publish/Subscribe communication paradigm facilitates binding between data sources and consumers of data [76], as the intermediate entity called “broker” manages registration and decouples entities in time and space. This improves system architecture flexibility (which deals with REQ#4 and REQ#2), as data sources do not have to be aware of the existence or number of data consumers and *vice versa*. Furthermore, some Publish/Subscribe communication protocols, such as the Message Queuing Telemetry Transport (MQTT), support reliable communication that includes retransmission of lost frames and acknowledge events (which guaranteed the delivery of information). Finally, the communication among the monitor and control system and the higher abstraction layers will be performed over standard network technologies such as TCP/IP (which allows using the existent communications infrastructure in the company).
  - Inter-systems services: One more time, the same philosophy applied in the case of “Interoperability” is valid here.

### 3.3. First Prototype Design

Once presented the reference architecture for our framework, we design and build the first minimum functional prototype based on it. In this section, we first evaluate the minimum features we must include in the prototype for being able to validate its usability. Later, we present the list of elements which have to be built and the physical architecture considered.

In manufacturing companies three basic elements are involved: workers, products and the workplace (usually a bench of some type). In some traceability solutions, such as fully automated solutions, only products and the workplace are taking into account. However, these kinds of solutions are commonly designed for heavy industry companies (such as automakers), where companies have very extensive work areas, lots of investment and production processes use high-volume products. In these scenarios, high-tech expensive deployments such as robotics (which may be part of both the traceability system and the production system), or high arches for automated reading of low frequency RFID tags (which can be used without risk of reading tags of wrong areas thanks to the breadth of the production area) are feasible solutions. In contrast, in small manufacturing companies products are made with small supplies, which does not require manipulation by robots (that sometimes cannot even be done), so that investment in traceability systems based on robotics is not justified (especially considering the resources these companies usually have). Furthermore, deployment of fully automated systems often requires large spaces, not always available in small companies (sometimes made up of less than five workers), so alternatives must be evaluated. In works on this issue [9,12], it is clear that the key to overcome this difficulty passes to monitor actions performed by workers without replacing them with robots, including heavy infrastructure in the work area or assigning additional tasks to operators which decline their productivity. Therefore, our prototype must include human interaction, and workers have to be incorporated into the system in the most appropriate way.

Products, as considered in the TF4SM framework, will be provided with a NFC tag which identifies each one unequivocally. Later, this NFC tag must be detected when products are placed on the work table and when they are being manipulated by workers, so both elements (workers and work areas) have to be provided with NFC reader capability. For the operators, we designed a cybernetic glove with only NFC reader capability as the objective was to create a very light wearable device, which does not impede work, and for which ”pay attention once placed” is not necessary. For the workspace, NFC reader capability was not enough (we designed a cybernetic surface with four different reader zones), so four types of LED are included to notify the user of the progress in the process.

**Figure 3:**
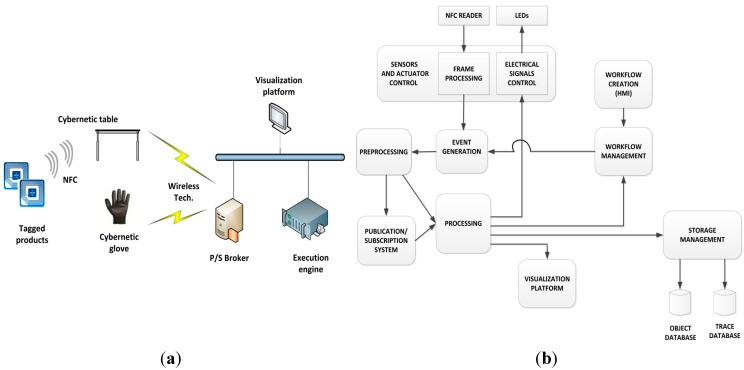
(**a**) Designed TF4SM physical architecture (**b**) Designed TF4SM functional architecture.

Previously described devices complete the cybernetic devices layer. Although in the general case layers can be implemented in different systems, in this first prototype we implement them into the execution engine to orchestrate the whole system. In order to enable human-system interactions at the top level, a visual application running in a visualization platform (or human-machine interface) is also provided. Through this platform, the state of the production process can be monitored and workflows and tasks can be defined (being also able to consult their compliance). Then, the final prototype’s physical architecture can be seen in Figure 3a.

As Figure 3a shows, another element has been included: a Publish/Subscribe broker. This element allows the spatial and temporal decoupling between publishers and subscribers and, in our system; it avoids having to reconfigure the rest of devices each time a device goes on or off. However, as this element belongs to the networking and communications plane and was not the focus of this work, it was omitted (an in-depth discussion about this subject can be found in articles, such as [77]). A functional architecture can be also considered (Figure 3b). First, we developed the NFC reader capability and the LED handling capability. These modules must be controlled from a component capable of extracting useful information from the NFC frames and capable of generating the electrical signal needed for turning on (or off) the LEDs. The cybernetic table and the cybernetic glove both support these capabilities. The event generation capability was later distributed among all the cybernetic devices and the execution engine. Depending on the system’s state at a particular moment any of these components can generate an event (see Section 4.2). Once an event has been generated, an evaluation step (executed in the same device where the event was produced) decides if the event must be published (using the Publish/Subscribe system) or any action must be directly executed (both options may be necessary). All the components in the prototype (cybernetic devices, execution engine and visualization platform) have processing capability (so they form a pervasive computing scheme). The processing step can cause a change in visualization platform, in LED state, a workflow update and/or an operation over the databases (in Section 4.1 the storage capacity of the system is described in detail). Both workflow management and storage management capabilities belong to the execution engine. Finally, depending on the workflow defined in the human-machine interface (HMI, also implemented in the execution engine), a workflow update can generate a new event, which performs the same cycle described.

## 4. System Construction

This section explains the implementation of the solution that was designed following the TF4SM framework, and its deployment in the application scenario.

### 4.1. System Implementation

As we have said in Section 3.3, the system must be made up of the following four elements: a cybernetic glove, a cybernetic table, an execution engine which communicates with cybernetic devices by means of a byte-oriented protocol, and a visualization platform displaying the status of the monitoring system and process execution.

One of the most important components in the designed system is the cybernetic glove. Even workers monitoring the system should increase process efficiency. However, human behavior is complex and sometimes random. Because of this, false measurements, erroneous events and similar mistakes can appear in the system if a cybernetic glove alone is considered (for example, as we will see later, if in the production process the glove passes near a tag it might be read, even when the object has not been picked up or used). In order to eliminate these measurement errors, more cybernetic devices are necessary (in this case we have selected a table because of the type of process considered). With the additional information provided by these additional devices, and using a proper algorithm (running in the execution engine), a more accurate picture of the current situation can be obtained.

**Figure 4:**
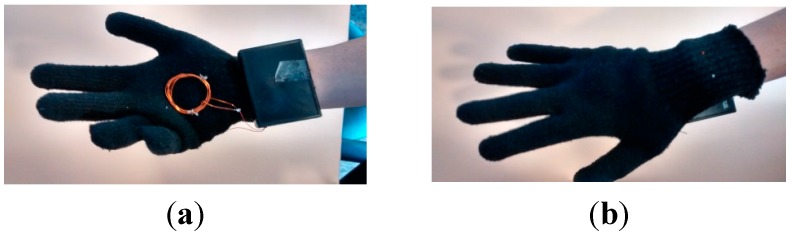
Example of hand movement with our cybernetic glove, (**a**) palm and (**b**) back sides.

Figure 4 shows our prototype for a cybernetic glove. The cybernetic glove consists of a plastic package placed on the front side of the wrist (so as not to impede movement), a galvanized copper coil to allow NFC communication with tagged cybernetic devices, and a synthetic textile support, whose properties do not significantly affect the magnetic field generated by the NFC module.

**Figure 5:**
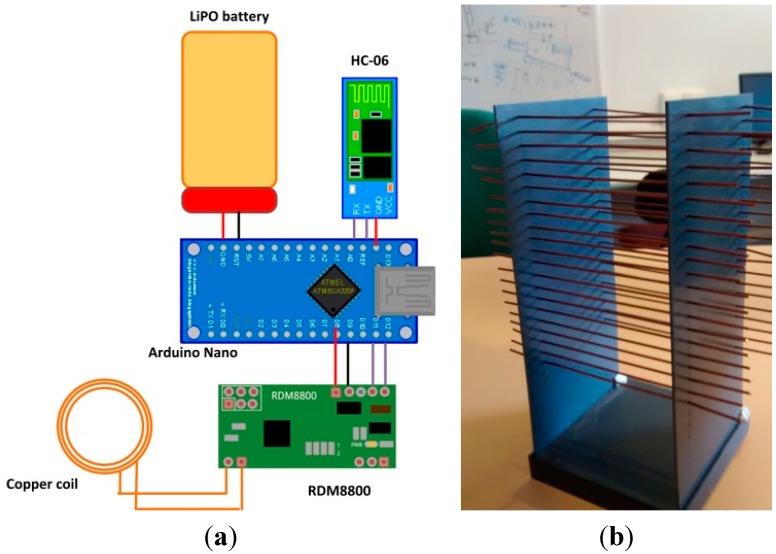
(**a**) Electronic scheme of the cybernetic glove; (**b**) Plastic holder graduated in height for measuring glove behavior.

In Figure 5a we present the electronic scheme of the cybernetic glove. As can be seen, five elements compose the glove: a LiPO battery, a galvanized copper coil as an inductive antenna, an RDM8800 NFC chip [78], an Arduino Nano platform, and an HC-06 Bluetooth slave module. The cybernetic table is also provided with four different types of LED.

A brief explanation of the function of each element follows:

- The LiPO battery has a capacity of 850 mAh and 3.7 V. As the total current consumption of the glove is 140 mA, the glove has the capacity to operate for about 6 h;
- The galvanized copper coil acts as an inductive antenna, resonant at NFC frequency (13.56 MHz);
- The RDM8800 NFC chip receives physical signals from the antenna, and produces a data frame encapsulating, among other fields, the identifier of the tagged cybernetic device that is in contact with the glove. The output interface is UART-serial type (at 9600 bauds);
- The Arduino Nano platform receives data from RDM8800 by UART and extracts the tagged cybernetic device’s identifier. Finally, it encapsulates the identifier in an application protocol message and transmits the message by a second UART at 19,200 bauds;
- The HC-06 Bluetooth slave module receives data by UART at 19,200 bauds and transmits them through a Bluetooth 3.0 interface;
- The table’s LEDs are used as actuators to notify the user of different types of events.

**Figure 6:**
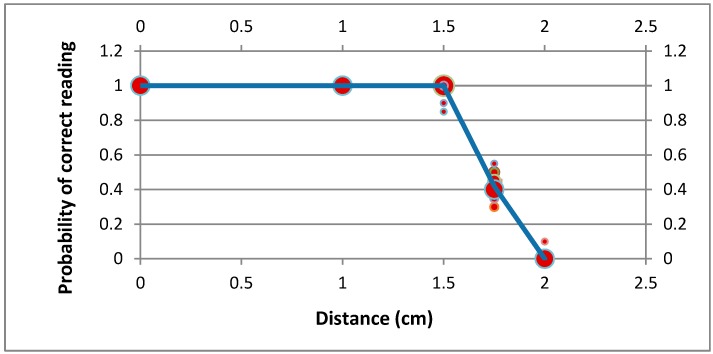
Evolution of the detection probability based on distance between the glove and the tagged device.

Thanks to NFC technology, and controlling some of the antenna configurations, we can greatly reduce the distance at which the glove detects the presence of a tagged cybernetic object (see Figure 6). Thus, we can say that the glove performs readings by contact.

Despite this, it is necessary to consider some limitations. On the one hand, metallic objects cannot be considered due to their capacity to affect magnetic fields. On the other, as the magnetic field generated by the NFC reaches a maximum distance of 1.5 cm and only one device can be detected at once by the glove, we must guarantee that only one tagged cybernetic device is less than 1.5 cm from the glove.

In order to measure the data, from which we prepared Figure 6, we used a plastic holder with several grids, separated (from each other) vertically by 0.5 cm (see Figure 5b). The experimental procedure was conducted as follows:

- Five points were selected in height: 0 cm, 1 cm, 1.5 cm, 1.8 cm and 2 cm;
- For each point in height, a set of 10 measurements were made. The probability of correct reading for each point was calculated by means of Laplace definition Equation (1):
  (1) $$P(correct\;reading)=\frac{number\;correct\;readings}{number\;tries\;}$$
- The second step was repeated ten times, obtaining ten different values of the correct reading probability for each point in height. Figure 5 shows the clouds of points obtained, along with a piecewise linear fit (blue line) obtained by minimizing the mean square error. The bubble size indicates the number of times a given value has occurred.

**Figure 7:**
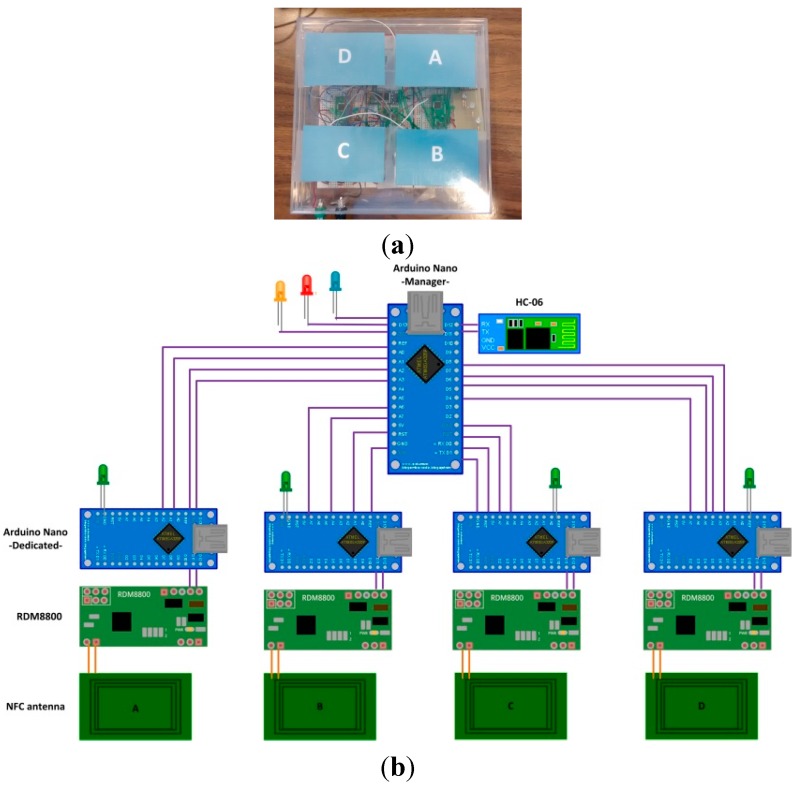
(**a**) Prototype of a cybernetic table; (**b**) Electronic scheme of the cybernetic table.

In Figure 7a we present our prototype for a cybernetic table. It is made of a plastic material (which does not interfere with electromagnetic fields) and on whose surface we have defined four detector areas.

In Figure 7b an electronic scheme of the table is presented. As can be seen, there are four dedicated microcontrollers (one for each detector area), and one manager microcontroller. When a dedicated microcontroller has data to transmit, it requests permission from the manager, and transfers the message using a physical protocol. This physical protocol consists of two lines: the permission line serves as output for manager and input for dedicated microcontrollers, and the solicitation line serves as input for manager microcontroller and output for dedicated units.

**Figure 8:**
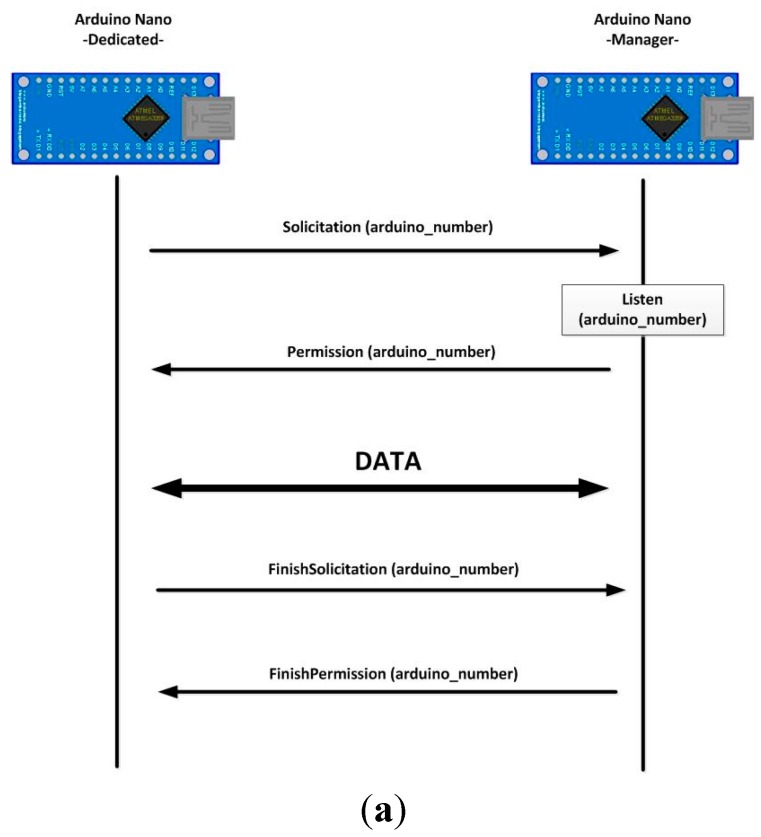

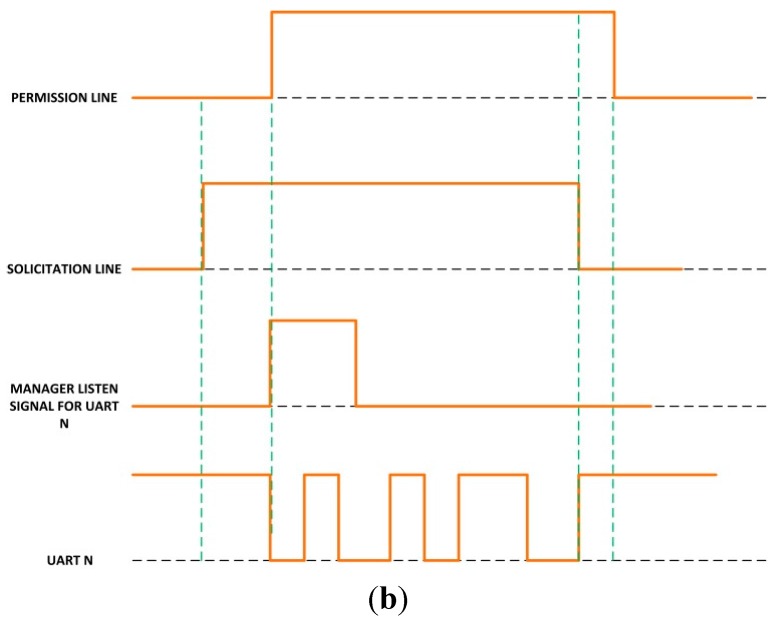
Physical protocol to allow communication between the manager Arduino and dedicated Arduinos, (**a**) Sequence diagram; (**b**) Chronogram.

The protocol is defined below and is also presented in Figure 8:

- When a dedicated microcontroller wants to transmit data using the Bluetooth module connected to the manager unit, it sets the solicitation line to high value;
- The manager unit recognizes this signal change and checks whether or not the Bluetooth module is in use. If it is not, it clears the Bluetooth buffers and deletes old transitions. It also sets the permission line to high value to indicate that it is expecting Bluetooth connections;
- When the dedicated microcontroller detects a high level in the permission line, it starts the data transmission. At the end of the transmission, it also sets the solicitation line to low level;
- Finally, when the solicitation line changes to low level, the manager microcontroller also sets the permission line to low level again.

**Figure 9:**
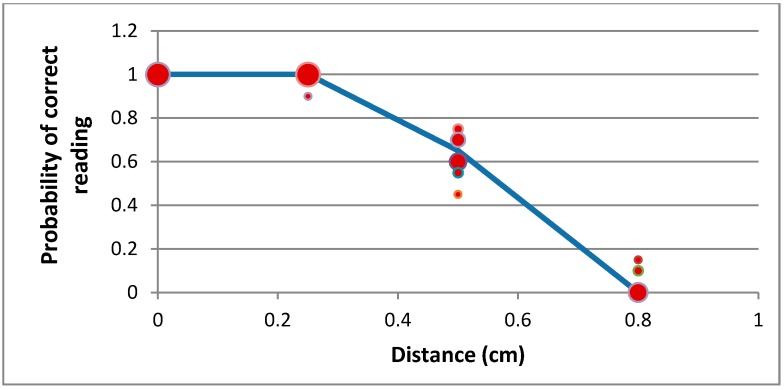
Evolution of the detection probability based on the distance between the table and the tagged device.

Additionally, the maximum distance at which a tagged device is detected by the table is much shorter than the corresponding distance in the glove (see Figure 9). As can be seen, this maximum distance is, in this case, 2.5 mm. To obtain this result, the same procedure explained for Figure 6 was followed (although, in this case, only four points in height were considered).

**Figure 10:**
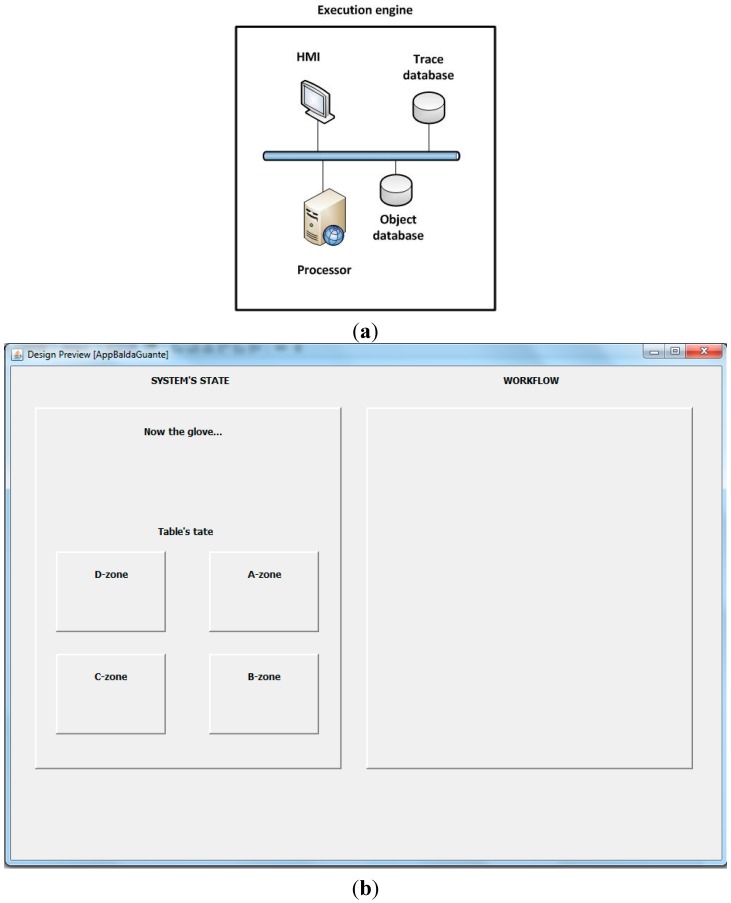
(**a**) Functional decomposition of the execution engine; (**b**) Visualization platform.

Figure 10a shows the functional decomposition of the execution engine. As can be seen, four elements can be characterized (see Section 3.3 and Figure 3b). First, there is an HMI through which users can define the system’s behavior and workflows. Second, it includes a processor where workflow management, storage management, event processing and generation and system coordination are executed. Finally, two databases for information storing are also included. The first one stores the traces generated by the system in order to be able to recover past executions. The second one stores all the information about cybernetic devices, users and tagged objects (such as ID, logical name and actions allowed). All the elements that make up the execution engine are built using Java technologies, such as JavaX Bluetooth libraries and hsqlDB manager.

As we have explained in the previous sections, the execution engine has workflow and storage management capabilities. Thus, it will usually be informed about all the events in the system. However, as we have said in Section 3 and will see in Table 2 and Table 3, our TF4SM framework (and consequently this first prototype) allows distributed event processing. Each event may be processed on the same device where it is generated (totally or partially), or it can be transmitted toward remote components. It is also possible to perform both actions.

Other capabilities, such as signal power or signal quality measurements, are considered to detect when a device is entering or leaving the system (this way the system will adapt automatically).

The visualization platform consists of a Java graphical application on which the system state and workflows advances are constantly shown (Figure 10b). Finally, we must describe the byte-oriented application protocol that allows communication among the different cybernetic devices and the execution engine. A byte-oriented (also called character-oriented or text-oriented) protocol is a communications protocol in which full bytes are used as control codes. The main advantage of these protocols is that they can be directly understood by humans.

In many CPS scenarios [79,80], other technologies such as XML or JSON are preferred, as they are also efficient, human readable and can also be transmitted via HTTP. However, this is not possible with the selected hardware (which only has 2 KB of SRAM and 30 KB available in EEPROM). First, specific and heavy libraries are necessary (because Arduino is not designed for parsing XML or JSON documents). Nevertheless, the EEPROM space is almost full with Arduino libraries (which includes String library), Publish/Subscribe protocol code and NFC processing code, so it would be quite impossible to add any additional library. Second, the control characters needed in XML or JSON are more than the ones used in a specific protocol, so communication slows down in these cases (We used virtual communication infrastructures and an 8 MHz processor). And, finally, a rigid message structure allows processing the messages while they are being received, so not all the characters must be stored, and the available SRAM needed is smaller. Therefore, we have designed a specific byte-oriented application protocol for this scenario.

**Figure 11:**
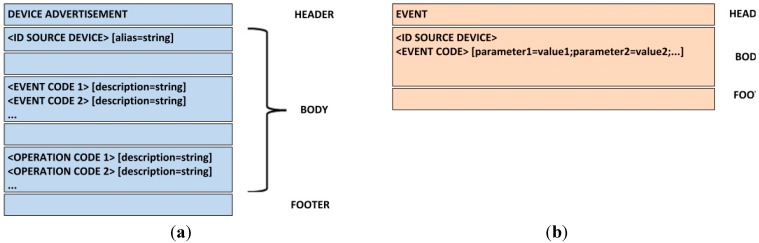
Scheme of protocol messages; (**a**) DEVICE ADVERTISEMENT message; (**b**) EVENT message.

In this case, only two different messages are considered: DEVICE ADVERTISEMENT message and EVENT message (Figure 11).

When a new cybernetic device (uniquely identified throughout the world by its Bluetooth MAC address) is connected to the prototype, the first thing it must do is to inform about the events it can generate (for example, new tagged-object detected) and the operations it can execute (such as blinking a LED). This way, the execution engine (which will be subscribed to the appropriate topic) can present that information on the HMI, and users are able to use them in their workflows and processes. This way the proposed prototype is adapted to the arrival of new devices and is adaptable to use new capabilities provided by the cybernetic device. This information is sent via a DEVICE ADVERTISEMENT message. The message structure is as follows:

- In the header line, we must indicate the message type: Device Advertisement;
- In the first line of the body, we must place the identification of the new device, followed by a blank space and (optionally) an alias which will appear on the HMI;
- Then, an empty line indicates the beginning of the list of events the device can generate. In each line, first the event code must be placed (see Section 4.2). Later, optionally and separated by a blank space, a description of the event including its parameters, meaning or any other information can be incorporated;
- A new empty line indicates the end of the list of events, and the beginning of the list of actions the device can execute. The list format is the same as in the previous case;
- The message ends with an empty line.

Being strict, as our prototype is only going to have two types of devices (cybernetic gloves and cybernetic tables), a manual configuration of these elements could be done, and the ADVERTISEMENT message is dispensable. However, in order to verify the full performance of the TF4SM framework (including the initial configuration) we have chosen to consider this transaction. An EVENT message is sent each time a cybernetic device generates an event, and this event must be notified to another device. The message structure is as follows:

- In the header line, we must indicate the message type: Event;
- In the first line of the body, we must place the identification of the device which notifies the event;
- In the second line the event code (see Section 4.2) must be placed first. Then, after a blank space, it is possible to place as many parameters as required in the event, following the format “parameter = value” and separated from each other by semicolons. The carriage return marks the end of the parameter list;
- The message ends with an empty line.

### 4.2. Process Model

Having described the physical implementation, in this section we now present how workflow definition and verification work in our prototype and, in general, how a TF4SM-based system operates.

In our prototype, we consider a workflow as a collection of states related to each other by transitions, which are executed when one or various events occur (as in a finite-state machine). Examples of workflows are the inventory and the manufacturing processes explained in the motivation scenario. Additionally, workflows are considered logical objects, so they encapsulate their own attributes and methods (such as the owner or the date of the last execution).

In each state two activities are executed, one when entering the state (entry activity) and another when exiting (exit activity). The problem is that the activities that are usually described in a workflow consist of several actions that cannot be run directly on cybernetic devices, but should be modeled using the operations they offer. An example of activity is the production task explained in the motivation scenario. Activities encapsulate their own attributes and methods (such as the activity number).

We define an activity as an ordered collection of atomic operations. One operation can only be executed if all the previous operations have been successfully completed. As activities are the smallest logical object, if one atomic operation fails, the whole activity fails. Finally, we call atomic operations those operations which can be executed only with one interaction in the prototype (for example, picking up a tagged-object, turning on a LED, *etc.*). Atomic operations match operations announced by cybernetic devices in their DEVICE ADVERTISEMENT message. Table 2 presents the atomic operations currently supported in our system.

**Table 2:** Atomic operations currently supported in the TF4SM.

Operation	Source	Description
LED_ON	Cybernetic Table	Turns on the LED specified in the *identification* parameter. If *durationTime* value is not included, LED remains on indefinitely. Otherwise, LED is on during *durationTime* seconds. If the LED is already on, nothing happens.
LED_OFF	Cybernetic Table	Turns off the LED specified in the *identification* parameter. If *durationTime* value is not included, LED remains off indefinitely. Otherwise, LED is off during *durationTime* seconds. If the LED is already off, nothing happens.
TAG_PUSH_GLOVE	Cybernetic Glove	Turns on the glove’s NFC interface. If any parameter is included, the operation finishes when any NFC tag is read. If *durationTime* value is specified NFC interface stays on during *durationTime* seconds. If *identification* parameter is included, NFC interface stays on until the tag with the specified ID is read. It is possible to combine *durationTime* and *identification* value to obtain more complex behaviors.
TAG_PUSH_TABLE	Cybernetic Table	Turns on the table’s NFC interface associated with *activeZone* area. If any parameter is included, operation finishes when any NFC tag is read. If *durationTime* value is specified NFC interface stays on during *durationTime* seconds. If *identification* parameter is included, NFC interface stays on until the tag with the specified ID is read. It is possible to combine *durationTime* and *identification* value to obtain more complex behaviors.
TAG_POP_GLOVE	Cybernetic Glove	Turns off the glove’s NFC interface. If any parameter is included, operation finishes when the NFC tag that is being read is withdrawn or if there is none, immediately. If *durationTime* value is specified NFC interface stays on during *durationTime* seconds and then turned off.
TAG_POP_TABLE	Cybernetic Table	Turns off the table’s NFC interface associated with *activeZone* area. If any parameter is included, operation finishes when the NFC tag which is being read is withdrawn, or, if there is none, immediately. If *durationTime* value is specified NFC interface stays on during *durationTime* seconds and then turned off.
SHOW_MESSAGE	Visualization platform	Shows on visualization platform a pop-up window, where the content of *message* value is printed.

Besides the atomic operations, as we mentioned in Section 3.3, cybernetic devices can also generate, process and receive different types of events. In an evaluation step, it is decided whether or not one event is processed in the same device where it is generated, or transmitted by means of an EVENT message to other devices (to execution engine, for example, in order to update workflow state). Table 3 presents the events that are currently supported in our system.

**Figure 12:**
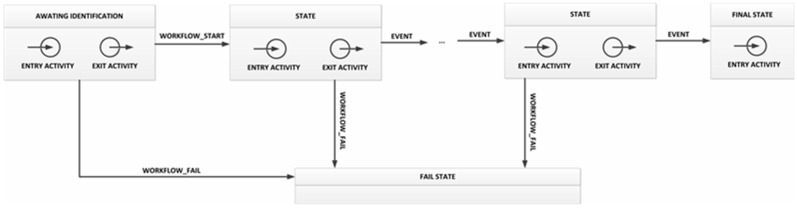
General workflow format in the TF4SM-based prototype.

Considering the motivation scenario (see Section 3.1), although various workflows can be executed in parallel (*receiving process* and *inventory process*), activities in each workflow such as the *production task* can always be executed sequentially. We simplify our process design and support only linear workflows. In this scheme, only one set of events allows advancing to the next state, and any other set causes the workflow to fail (see Figure 12).

Finally, three states are mandatory in all the workflows defined in this first prototype. First, in all workflows *the fail state* must be included. When a WORKFLOW_FAIL event is triggered, workflow transits toward *fail state* from any other state. Second, the *final state* is also mandatory.

**Table 3:** Events currently supported in the TF4SM.

Event	Source	Description
TAG_PUSH_GLOVE	Cybernetic Glove	This event is triggered when an NFC tag is detected by the cybernetic glove. In *identification* parameter, the read tag’s ID must be indicated.
TAG_PUSH_TABLE	Cybernetic Table	This event is triggered when one NFC tag is detected by the cybernetic table. The *activeZone* value includes the reader area where the tag has been detected and in *identification* parameter, the tag’s ID must be indicated.
TAG_POP_GLOVE	Cybernetic Glove	This event is triggered when an NFC tag previously detected by the cybernetic glove is withdrawn. In *identification* parameter, the withdrawn tag’s ID must be indicated.
TAG_POP_TABLE	Cybernetic Table	This event is triggered when one NFC tag previously detected by the cybernetic table is withdrawn. The *activeZone* value includes the reader area where the tag has been withdrawn and in *identification* parameter, the withdrawn tag’s ID must be indicated.
WORKFLOW_START	Execution engine	This event is triggered when a workflow has been recovered by a user and must start. In *identification* parameter the workflow ID is indicated.
WORKFLOW_END	Execution engine	This event is triggered when a workflow has finished successfully. In *identification* parameter the workflow ID is indicated.
ACTIVITY_COMPLETE	Execution engine	This event is triggered when an activity has finished successfully. In *identification* parameter the activity ID is indicated and, in *parentIdentification*, the workflow’s ID to which this activity belongs is included. This event will allow not only store traces of atomic operations and activities.
ACTIVITY_FAIL	Execution engine	This event is triggered when an activity has failed. In *identification* parameter the activity ID is indicated and, in *parentIdentification*, the workflow’s ID to which this activity belongs is included.
WORKFLOW_FAIL	Execution engine	This event is triggered when a workflow has failed. In *identification* parameter the workflow ID is indicated.
WORKFLOW_NEW	Execution engine	This event is triggered when a new workflow is created in the execution engine. In the *workflow* parameter, the new workflow is codified using bytes.

This state represents the completion of the state machine and does not have exit activity. Third, the *awaiting identification* state must be considered an initial state. This state represents the workflow’s starting point, and always has to be defined as follows:

- Entry activity: first a SHOW_MESSAGE operation must be executed in order to ask users to identify themselves. Then, a TAG_PUSH_GLOVE operation turns on the glove’s NFC interface, which is not deactivated until the users again uses his ID to close the state;
- As the execution engine subscribes to the corresponding topic (see Section 4.3), it will receive information on the user’s ID. The execution engine checks whether the workflow owner user is the same user that was identified. If so, a WORKFLOW_START event is generated;
- Transition: the workflow will execute the transition toward the next state when a WORKFLOW_START event is received;
- Exit activity: this activity is made up of only one SHOW_MESSAGE operation, to indicate to users that the workflow verification has started.

Although the process model described is a simplification, it matches the model used in a large number of manufacturing companies. For example in [81] the cigarette manufacturing process is described by workflows consisting on sequences of activities beginning at an initial activity and ending at a completion activity. Transitions between activities also are run when the associated trigger event occurs. For all this, the model can be considered valid to verify the usability of our prototype.

### 4.3. System Deployment

Our first TF4SM-based prototype was deployed as a laboratory prototype at the Technical University of Madrid. The scenario was built simulating the scenario we can find in a real small-sized manufacturing company.

In the object database we have registered 56 different objects and 36 different user profiles. In the execution engine we have developed only one workflow, made up of 10 different states (13, if we take into account the mandatory states describe above). Finally, in this prototype, devices have been configured as Table 4 shows.

**Table 4:** Devices’ configuration.

Device	Configuration
Cybernetic glove	All the events generated in the glove are directly published without been processed.
Cybernetic table	The TAG_PUSH_TABLE event turns on one green LED. The TAG_POP_TABLE event turns off the same green LED. The WORKFLOW_START event turns on an orange LED. Both TAG_PUSH_TABLE and TAG_POP_TABLE events are also published. The WORKFLOW_END event turns on a blue LED for 10 seconds, and the WORKFLOW_FAIL does the same with a red LED.
Visualization platform	It is subscribed to all the topics that are used to update the platform.
Execution engine	It is subscribed to all the topics that are used to update the workflow, and also to publish all the events it generates.

## 5. Experimental Validation

This research paper attempts to answer the following research questions:

1. Would the time response to inefficiencies improve by deploying a system based on our TF4SM in companies?
2. Is it possible to reduce the number of inefficiencies in productive processes using a TF4SM-based system?

An experimental validation was carried out in order to address these research questions. In this experimental validation two different experiments were executed. In the first one, the response time to inefficiencies (specifically, product shortages) is qualitatively compared in our proposed protoype with a traditional tag-based traceability system (similar to the one described in [23]). In the second one, we compare a traditional tag-based traceability system with our proposed TF4SM-base prototype, where notifications to users about process execution are activated for the number of inefficiencies by worker.

**Figure 13:**
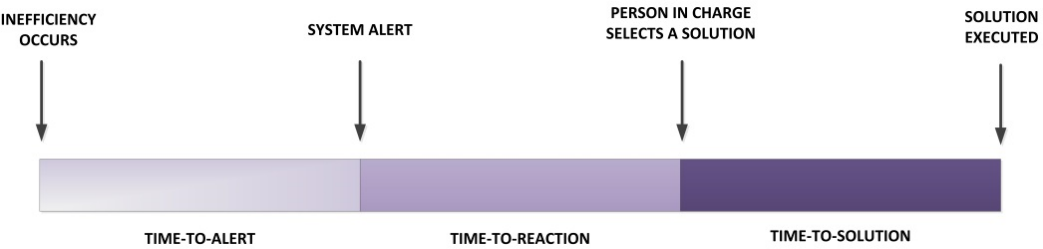
Response time to inefficiencies decomposition.

The total response time to inefficiencies may be grouped into three different times (Figure 13). The *time-to-alert* time starts when the inefficiency occurs and finishes when an alert is generated in the process monitoring system. Secondly, the *time-to-reaction* time starts when an alert is generated in the process monitoring system and finishes when the person in charge responds to the alert and identifies the solution. Finally, the *time-to-solution* time involves all the time needed to solve the problem previously identified.

One of the most important inefficiency types, in which response time is critical, is product shortages. If the amount of remaining product is not accurately controlled, it might not only stop production (such as when other types of inefficiencies occur), but also all the processes in which the product is needed (which can be most processes if it is a basic product).

In product shortages, the *time-to-solution* is independent of the monitoring process implemented as it is more related to the presence or not of automatic order systems. Consequently, we conducted an experiment to qualitatively compare a traditional tag-based traceability system with our prototype for the *time-to-alert* and *time-to-reaction* times in the case of product shortage.

Thirty six (36) people were involved in this first experiment. In our manufacturing scenario, these people were divided into two equal groups called the *TF4SM* and *TAG* groups. Both groups were isolated from each other. Within each group four different productive processes were assigned at random among the participants, and one additional person was in charge of solving the possible product shortages. 56 different products were located in the warehouse, but the amount of some of them was insufficient to finalize all the processes in execution.

In the *TF4SM* group all the products were on a cybernetic table that monitored the amount of remaining product. Each worker also wore our cybernetic glove. The person in charge was also provided with a mobile visualization platform for controlling the warehouse state.

In the *TAG* group, each worker had a tag-reader, which he had to pass over the product’s tag before removing one unit from the warehouse. There was also a control point where commercial retail control software runs, and where the person in charge could verify the products available in the warehouse.

Finally, an external observatory was placed in both groups to qualitatively evaluate the *time-to-alert* and *time-to-reaction* times.

In the second experiment, the number of inefficiencies was evaluated. In general, inefficiencies were due to three types of errors. Firstly, there were inefficiencies due to procedural errors. In these cases, an activity was not executed in the correct order, unfinished or some step had not been executed. Secondly, there were inefficiencies due to execution errors. In these cases, all steps of the activity were executed, but (at least) one of them had not been developed as expected (a badly tightened screw or badly applied paint were examples of such errors). Finally, there were inefficiencies due to other errors, such as power outages and shortages.

In traditional tag-based systems, all errors are considered equal, and were evaluated at the workflow’s end (in the so-called quality control point). However, our TF4SM-base prototype allows to provide users with real-time information on process execution and, therefore, it enables the possibility of notifying procedure errors as they occur. Thus, workers can correct their last action, thereby reducing the number of errors when the product is delivered for quality control.

To compare the inefficiencies in a manufacturing company where a traditional tag-based traceability system was deployed with the inefficiencies in a manufacturing company where our proposed TF4SM-base prototype was available, we conducted the following validation.

Thirty six (36) people were involved in this second experiment. In our warehouse scenario, these people were divided into two separate groups. *TF4SM* was made up of 20 people and *non-TF4SM* the remaining 16.

The *TF4SM* group went one by one to a cybernetic table where they had to run a 10-step workflow. Each user had 16 kinds of products, of which only nine had to be processed, and they wore our cybernetic glove. During the execution of the workflow, our prototype monitored the process in real-time and alerted workers by means of a red LED whether they had performed an action that was not provided. At any time workers could undo any action. At the end of the workflow, an expert validated the final product and, in case of inefficiency, he specified the number of errors and type.

The *non-TF4SM* group worked in a similar way, except that in this case; the prototype was turned off, leaving only as checkpoint the final quality control (as in most manufacturing processes).

## 6. Results

This section presents and discusses the results obtained in the experimental validation. Results are shown following the research questions defined in this paper. Table 5 summarizes the magnitude order of the *time-to-alert* and *time-to-reaction* times for the first experiment.

**Table 5:** Devices’ configuration.

System Employed	*Time-to-Alert* Time	*Time-to-Reaction* Time
Tag-based system	Less than 10 s	Between 1 and 3 min
TF4SM	Immediate	Less than 30 s

Some considerations should be given to Table 5. First, we verified that time response to inefficiencies improved by deploying our prototype. As the system required no consumables, the only expenditure was the initial investment, so we can say that we have achieved this improvement. Second, we can explore some reasons for this improvement. In respect to *time-to-alert* time, the need for human intervention in traditional tag-based systems causes, inevitably, higher delays. Operations, such as finding the tag or preparing the reader, can be made really fast, but automatic systems (such as the one TF4SM framework proposes) will always be much faster than humans. With respect to *time-to-reaction* time, the event monitoring independence from hardware (achieved in TF4SM framework) allows to develop mobile platforms that are permanently connected to the person in charge (who can respond to the emergency very quickly). In traditional tag-based systems, software commercial platforms require hard configurations and, in most cases, specific hardware to be deployed. Therefore, as in our experiment, in most companies there is a fixed control point that is periodically reviewed by managers. Depending on the review period, events may be processed faster or slower.

**Figure 14:**
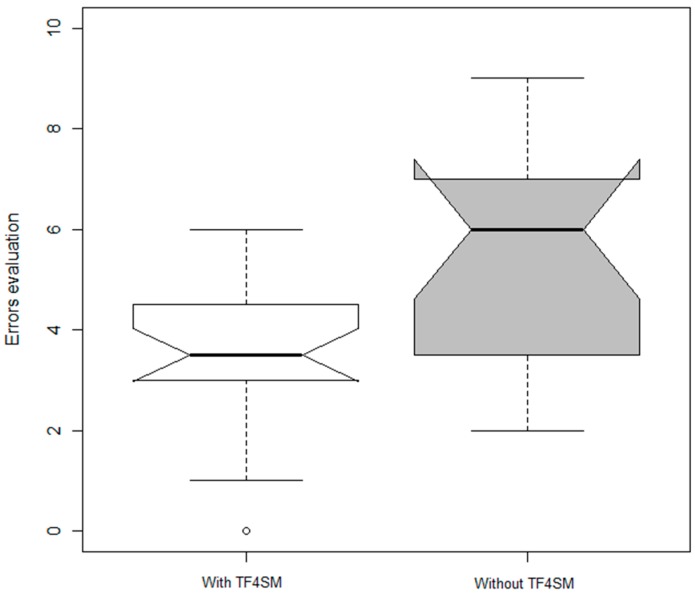
Response time to inefficiencies decomposition.

Considering the second experiment, Figure 14 shows two boxplot measuring the number of errors committed by the *TF4SM* group (median = 3.5, SD = 1.76) and the *non-TF4SM* group (median = 6, SD = 2.28).

As both boxplots have an overlapping area, a Mann-Whitney U test was conducted to confirm whether the use of our proposed TF4SM framework reduces the number of errors. The Mann-Whitney U test is a nonparametric test of the null hypothesis that two samples come from the same population against an alternative hypothesis, comparing the mean values of the two samples. It is used to evaluate if two different data populations are similar or different (higher or lower). The *p*-value indicates the significance level of Mann-Whitney U test. The results are positive and support the previous assertion within the expected significance, *p* < 0.005 (U-values omitted as the comparison results have relatively little importance).

As can be seen, our proposed TF4SM framework does not eliminate the inefficiencies, but it allows workers to make some mistakes (specifically, procedure errors) and gives them the opportunity to correct a production task, which, in the end, reduces the number of inefficiencies (answering in this way our second research question).

## 7. Conclusions

Traceability systems are rapidly becoming very important in manufacturing sector. Governments, costumers, workers among others ask companies for tighter controls in production processes. However, the complexity associated with manufacturing processes nowadays makes this task greatly complicated, especially for small companies. On the one hand, traditional traceability systems increase costs when logistics increases in complexity, and the necessary investments are not always acceptable for small companies. On the other hand, any type of completely automatic process monitoring or traceability system is widely used in industry, so companies are reluctant to implement such solutions (except in the case of proprietary solutions, something unattainable for small companies). Our Traceability Framework For Small Companies (TF4SM) fills this gap, by allowing real-time traceability and process monitoring through a flexible, open architecture (based on CPS NIST definition) capable of adapting to all types of manufacturing companies.

With TF4SM-based systems small manufacturing companies can monitor both workers’ actions and movement of the products at any given moment. This, together with the ability to use mobile platforms for system control, allows a significant reduction in the response time to inefficiencies obtained in our systems, compared to traditional tag-based traceability systems.

Moreover, the use of real-time monitoring enables the possibility of notifying users of their errors, allowing the workers to correct them (when it is possible). Thus, the number of inefficiencies in companies that implement TF4SM-based traceability systems is lower than in companies that use traditional traceability systems.
